# Biogenic synthesis of AgNPs employing *Terminalia arjuna* leaf extract and its efficacy towards catalytic degradation of organic dyes

**DOI:** 10.1038/s41598-020-66851-8

**Published:** 2020-06-15

**Authors:** Shani Raj, Hanwant Singh, Rohini Trivedi, Vineet Soni

**Affiliations:** 1 Laboratory of Plant Pathology, Department of Botany, University College of Science, Mohanlal Sukhadia University, Udaipur, 313001 Rajasthan India; 2 Plant Bioenergetics and Biotechnology Laboratory, Department of Botany, University College of Science, Mohanlal Sukhadia University, Udaipur, 313001 Rajasthan India

**Keywords:** Nanoscale biophysics, Environmental biotechnology, Environmental chemistry

## Abstract

In the present work, we demonstrated the biosynthesis of silver nanoparticles (AgNPs) by highly stable, economic and eco-friendly method using leaf extract of *Terminalia arjuna* (*T. arjuna*) and employing as a catalyst for the degradation of methyl orange (MO), methylene blue (MB), congo red (CR) and 4- nitrophenol (4-NP). The biosynthesis of AgNPs was visually validated through the appearance of reddish-brown color and further confirmed by the UV-spectra at 418 nm. The TEM and FE-SEM studies revealed the spherical shape of particles with size ranged between 10–50 nm. Face centered cubic crystalline nature of AgNPs was proved by XRD analysis. The negative value of zeta potential (−21.7) indicated the stability of AgNPs and elemental composition was confirmed by EDS. FT-IR analysis revealed the functional groups present in the plant extract trigger the biosynthesis of AgNPs. The AgNPs exhibited strong degradation of MO (86.68%), MB (93.60%), CR (92.20%) and 4NP (88.80%) by completing the reduction reaction within 20 min. The reaction kinetics followed the pseudo-first-order and displayed k-values (rate constant) 0.166 min^−1^, 0.138 min^−1^, 0.182 min^−1^ and 0.142 min^−1^ for MO, MB, CR and 4-NP respectively. This study showed an efficient, feasible and reproducible method for the biosynthesis of eco-friendly, cheap and long-time stable AgNPs and their application as potent catalysts against the degradation of hazardous dyes.

## Introduction

Nanotechnology deals with the synthesis and manipulation of particles ranging from 1–100 nm in size at one dimension^1^. From the decades, metal nanoparticles (NPs) are gaining more attention in the field of nanotechnology due to the higher specificity and activity than their bulk counterpart^2^. Metal NPs such as platinum, silver and gold have been broadly used as photonics, electronics, optical device, catalyst, biosensing and bio-labelling agents^3–10^. Among metal NPs, AgNPs have found appropriate claimants as antimicrobial and anticancer agents, wound healing, drug delivery system and waste water treatment^11–14^. In order to meet the demands of NPs, various physical and chemical methods have been exploited in recent years^15–19^. The disadvantages of the conventional methods of metal NPs synthesis are cost expensive, high energy input and use of highly toxic chemical and solvent which are environmentally harmful. To reduce the harmful effects and costliness researchers have attracted towards the development of biological methods for synthesis of NPs that employs living organisms such as algae, fungi, bacteria and plants^20–25^. Plants are rich source of phytochemicals such as polysaccharides, flavonoids, alkaloids, terpenoids, phenolics, saponins, amino acids, proteins and vitamins which act as reducing and stabilizing agents for the biosynthesis of NPs^26^. Biological synthesis of AgNPs using plant extract has become ubiquitous due to easy to access and high efficiency^27^.

The plant *T. arjuna* Wight and Arn. (Combretaceae) is found abundantly throughout Indian subcontinent, Sri Lanka, Burma and Mauritius. The plant is used to treat many ailments due to presence of various bioactive compounds like luteolin, arjungenin, terminic acid, arjunoside I, arjunoside II, arjunolic acid etc^28,29^.

Nowadays, harmful chemical substances such as organic dyes have become the root cause of water contamination. Organic dyes are widely used in various industries as colorant such as textile, paper, food, drug, cosmetics, leather and printing^30^. A bulk amount of industrial effluents along with organic dyes are often discharged into the water bodies without passable treatment^31^. These dyes and its derivatives are highly toxic, carcinogenic and non-degradable which causes numerous problems such as skin diseases, liver and kidney failure and also poisoning the nervous system of living organism^32^. As currently available methods for the removal of toxic substances from water bodies are costly and inefficient, therefore, it is highly essential to develop an eco-friendly and cost-effective alternative for the degradation of organic dyes from wastewater^33^. Recently, the catalytic reduction of organic dyes using biologically synthesized NPs received the attention of scientists due to the high potential of degrading organic dyes^34,35^. However, the method of synthesis of nanoparticles using plants is popular but the study of application of silver nanoparticles on treatment of dye effluent is limited. Hence, there is crucial need of more assessment and evaluation in this field.

In this work, a rapid synthesis of AgNPs via eco-friendly method throught the leaf extract of *T. arjuna* has been developed. The synthesized AgNPs were also characterized using TEM, FE-SEM, EDS, XRD and FT-IR. Further, the developed AgNPs have also been used as catalyst for the degradation of MO, MB, CR and 4-NP.

## Materials and Methods

### Materials

AgNO_3_ (Sigma-Aldrich, St. Louis, USA) and dyes MO, MB, CR, 4-NP and NaBH_4_ (HiMedia, India) were procured from Pvt. Ltd. New Delhi (India). Healthy leaves of *T. arjuna* were collected from Madan Mohan Malviya Ayurveda College Udaipur, Rajasthan, India (24.5854° N, 73.7125° E). Sterilized deionized water (DIW) was used during the whole experiment process.

### Preparation of the plant extract

The leaves were washed thoroughly with double distilled water and shade dried for 2 weeks at room temperature. To make a fine powder, dried leaves were pulverized by mixture grinder. Thereafter, 1 g powder was mixed with 100 ml of boiled DIW and stirred on hot plate for 15 minutes. After that the extract was filtered through Whatman No. 1 filter paper (HiMedia). The filtrate was kept at low temperature (4 °C) for further use.

### Synthesis of Ag nanoparticles

1 mM of AgNO_3_ was prepared into sterilized deionized water and mixed with plant extract in ratio 95:5. The overall reaction was carried out in low light condition to minimize the photo-activation of AgNO_3_. After few minutes, solution was turned into reddish-brown color which indicates the reduction of AgNO_3_ into AgNPs. Further the solution was characterized by spectrum using UV- visible spectrophotometer (SPECORD 200 Analytik Jena, Germany). The reaction mixture was centrifuged at 15000 × g for 20 min at 4 °C. The pellets were collected and dried in the oven at 40–45 °C to obtain AgNPs.

### Characterization of Ag nanoparticles

UV–visible spectroscopy was done to study the bioreduction of AgNO_3_ to AgNPs. The reaction mixture was analyzed at scanning range 300–700 nm, 1 nm resolution and 1 cm path length with DIW as reference. DLS (Malvern, UK) was performed for the zeta potential of synthesized NPs at 25 °C at a scattering angle of 90°. FTIR spectroscopy (Bruker, US) was used to analyze functional groups which are involved in reduction of AgNO_3_ into AgNPs. The transmittance was recorded at 500 to 4000 cm^−1^. XRD (X-ray diffractometer, Ultima IV, Rigaku, Japan) was used to obtain diffraction pattern of AgNPs with Cu Kα radiation (λ = 1.54 Å) between 20° to 90° (2θ range). Electron microscopy was carried out to morphological study of the synthesized NPs using Transmission electron microscopy (TEM-Tecnai G2–20, USA) and Field Emission Scanning Microscopy (FESEM, JEOL SM-7600F Japan) coupled with X-ray energy dispersive spectrometer (Oxford EDS system). TEM analysis was done by ultrasonically dispersal of AgNPs powder in EtOH. Further, the one drop of the dissolved sample was placed on a Cu grid and allowed to evaporate at room temperature and operating at 200 kV with a resolution point of 2.04 nm.

### Catalytic degradation of dyes

Catalytic potential of synthesized AgNPs was evaluated by degrading different carcinogenic organic dyes (MO, MB, 4-NP and CR) in the presence of NaBH_4_. Before degradation process the synthesized AgNPs were sonicated using ultra prob sonication for few minutes to make aqueous colloidal suspension. For reduction, colloidal suspension of AgNPs and NaBH_4_ was mixed with aqueous solution of dyes. The detail of the concentration and volume of reaction mixture is given in Table 1. The reduction process was carried out in 4 mL quartz cuvette and time-dependent reduction time was observed by recording absorption spectra in time interval of 1 min. Reaction mixture without AgNPs was used as reference and pseudo-first-order kinetics was analyzed to evaluate the rate constant as per following equation:

1 $$\mathrm{ln}({{\rm{A}}}_{{\rm{t}}}/{{\rm{A}}}_{0})=-\,{\rm{kt}}$$

**Table 1:** Detail of experiment scheme for catalytic degradation of dyes.

Dye	Dye’s conc.	Volume of dye	Volume of H_2_O (DI)	NaBH_4_ (0.05 M)	Volume of AgNPs (0.05%)	Reduction time (min)	Rate constant (k)
MO	1 mM	200 µL	1.8 mL	0.990 mL	10 µL	14	0.166 min^−1^
MB	1 mM	100 µL	1.9 mL	0.980 mL	20 µL	19	0.138 min^−1^
4-NP	5 mM	50 µL	1.45 mL	0.580 mL	20 µL	15	0.142 min^−1^
CR	1 mM	400 µL	1.6 mL	0.980 mL	20 µL	14	0.182 min^−1^

**Figure 1:**
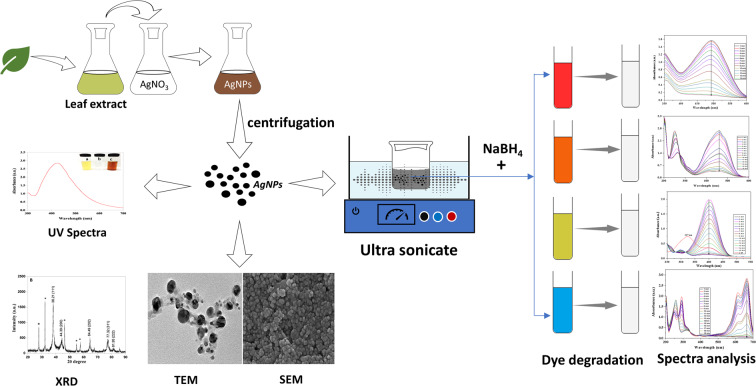
Schematic representation of the synthesis of AgNPs and degradation of dyes.

The % degradation of the dyes was estimated through the following equation

2 $${\rm{Percent}}\,{\rm{degradation}}=\frac{{A}_{0}-{A}_{t}}{{A}_{0}}\times 100$$

where, A_0_ is the initial absorbance of dye, A_t_ is the absorbance of dye at time t and k is the rate constant. The whole reaction of degradation was processed at room temperature. Figure 1 shows the over view of AgNP synthesis, characterization and dye degradation.

## Results and Discussion

**Figure 2:**
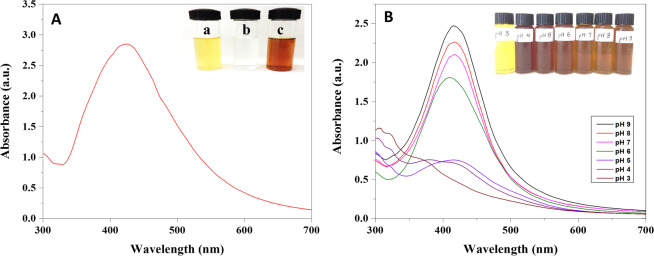
(**A**) UV-visible spectra of biosynthesized AgNPs [inset (a) Plant extract (b) AgNO_3_ solution (c) AgNPs] (**B**) UV-visible spectra of AgNPs at different pH.

A change in color from yellow to reddish-brown was observed upon the addition of plant extract into aqueous AgNO_3_ solution, which clearly indicates the synthesis of AgNPs. The change in color was due to surface plasmon resonance (SPR) excitation which was examined using UV-visible spectrophotometer. The SPR absorption band is due to collective vibration of free e^-^ of metal NPs in resonance with light wave^36^. Further, the formation of AgNPs was confirmed by the appearance of a characteristic peak at 418 nm (Fig. 2A). In this experiment, effect of different pH (3 to 9) on reduction of AgNO_3_ into AgNPs was assessed by UV- visible spectroscopic analysis (Fig. 2B). The size and shape of the AgNPs were influenced by the varying pH value. In the biological synthesis of AgNPs, pH changes the electrostatic repulsion of biomolecule and capping agent present in the solution resulting in changing their binding and reduction ability to metal ions^37,38^. It was indicated in Fig. 2B that with the gradual increase in pH the color of the solution (inset Fig. 2B) as well as absorbance intensity also increased but in case of pH 3 and 4 no characteristic peak was observed. From neutral to slightly alkaline pH sharp absorbance peak was observed which may be due to the ionization of the phenolic groups present in plant extract indicated rapid formation of AgNPs^39^. The formation of large size and aggregation of the AgNPs at acidic pH could be due to the electrical charge of the biomolecule which repels the anions present in the reaction solution^40^. By the result of this experiment, it can be observed the more reduction of Ag^+^ ion at pH at 9 is optimum condition.

**Figure 3:**
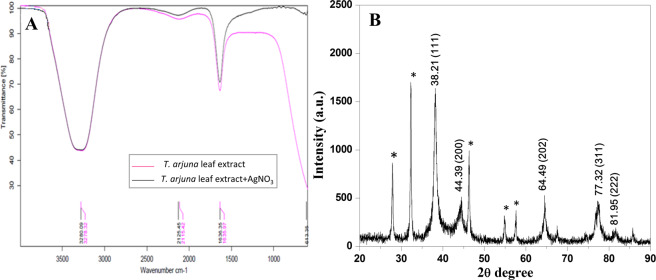
(**A**) FT-IR spectra of plant extract and AgNPs after reduction (**B**) XRD pattern of biosynthesized AgNPs.

Figure 3A shows the FTIR spectrum of leaf extract of *T. arjuna* before and after bioreduction of AgNO_3_ into AgNPs. FTIR spectrum analysis was done to recognize the possible biomolecules present in the leaf extract which are responsible for the reduction of the Ag^+^ ions into Ag° and capping of AgNP. In the spectrum low peak at 613 cm^−1^ is correspond to CI stretching of halo compound. The medium band at 1636 cm^−1^ is correspond to N-H stretching of conjugated amine and medium band at 2125 cm^−1^ correspond to C-N stretching of any alkyne N = C = S. The strong and broad band at 3280 cm^−1^ correspond to O-H stretching of stretching of H- intramolecular bonded alcohols and phenols and hence, the main component present in the leaf extract of *T. arjuna* such as leucoanthocyanidins, arjunetin and hydrolyzable tannins are responsible for the reduction of AgNO_3_ into AgNPs and capping of AgNPs^41^.

XRD crystallography was done to reveal the crystalline nature of the synthesized AgNPs. The XRD pattern showed the Bragg’s reflection plane in the 2θ range between 20–90°. The diffraction peaks at 2θ = 38.21°, 44.39°, 64.49°, 77.32° and 81.95° are corresponded to (111), (200), (202), (311) and (222) Miller indices of AgNPs, respectively Fig. 3B, which interpreted for the face-centred cubic structure of the AgNPs^42^. The broad peaks indicate the small crystalline size of nanoparticles. The resulted Bragg’s diffraction peaks are corroborated with database of Joint Committee on Powder Diffraction Standard of Ag (JCPDS card No. 04–0783). The similar findings of Bragg’s refection for AgNPs are also reported^43–45^. The sharp peaks are shown in Fig. 3B might have due to capping agent which stabilize the AgNPs and few unassigned peaks (*) that might be thought due to the crystallization of the biomolecule on the surface of the AgNPs^46^. The lattice constant was calculated from diffraction pattern α = 4.07 Å and d spacing 2.03 Å of synthesized AgNPs.

The mean size of the AgNPs was determined as per following Scherrer equation:

3 $${\rm{D}}={\rm{k}}{\rm{\lambda }}/{\rm{\beta }}\,\cos \,{\rm{\theta }}$$

where, D is the average crystalline size (Å), k is a constant equal 1, λ is X-ray radiation (λ = 1.54 Å) source, β is the angular line full width at half maximum (FWHM) and θ is the Bragg angle. The average size of AgNPs was noted as 18.11 nm.

**Figure 4:**
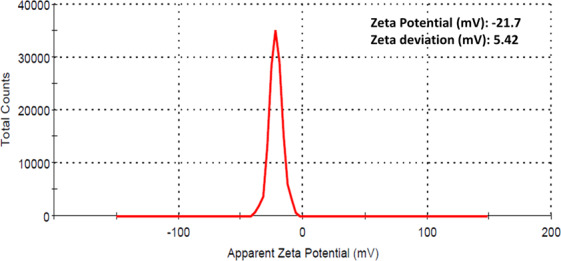
Zeta potential of biosynthesized AgNPs.

DLS studies were utilized to examine the surface zeta potential and dispersity of the synthesized AgNPs in aqueous colloidal solution. The PDI value of AgNPs was 0.963 **(**Fig. 4**)**. In the Fig. 4 negative zeta potential was observed at −21.7 mV and zeta deviation was 5.42 mV. The high negative value of zeta potential causes repulsion among nanoparticles to prevent agglomeration^47^. The size of nanoparticle measured larger than TEM, SEM and XRD due to the measured size included biomolecule and water layer covering the surface of nanoparticles^48^.

**Figure 5:**
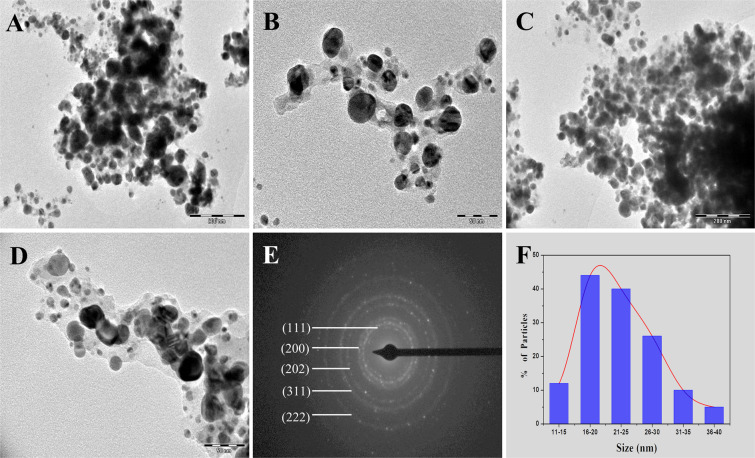
TEM micrograph of biosynthesized AgNPs with different magnification (**A–D**), SAED pattern (**E**) and Size distribution histogram of AgNPs (**F**).

**Figure 6:**
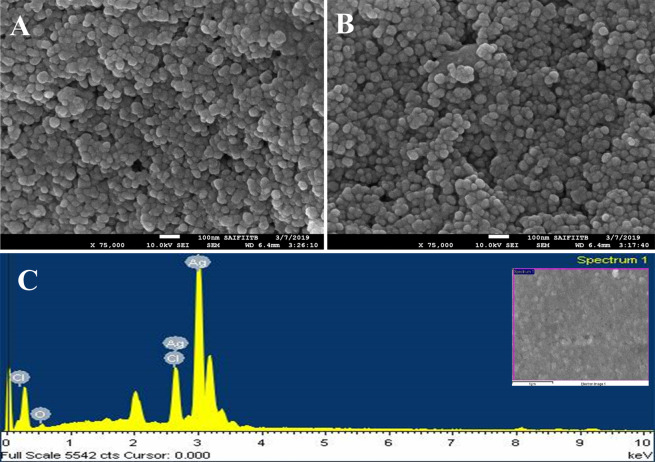
SEM images of biosynthesized AgNPs (**A**,**B**), SEM-EDS spectrum analysis (**C**).

FE-SEM and TEM were carried out to examine morphology of particle surface and size of plant-based produced AgNPs. TEM images (Fig. 5A–D) shows the particles are spherical and small groups of particles were also observed due to agglomeration during sample preparation. Figure 5E displays the Selected Area Electron Diffraction (SAED) pattern of the AgNPs which indicated the pointed diffraction spot in ring arrangement from inner to outer related with (111, 200, 202, 311, 222) lattice reflections of Ag^49,50^. FE-SEM study reveals that the particles are uniformly spherical in shape and the rough surface of the particles coated with organic layer which act as capping agents (Fig. 6A,B). The size of the particles was calculated by the TEM and FE-SEM analysis, which are mainly in range between10–50 nm. Highest percentage of particles showed the size range between 16–20 nm as displayed in size distribution histogram (Fig. 5F**)**. In addition, the chemical compositions of the synthesized AgNPs were revealed by EDS spectrum analysis, as shown in Fig. 6C. The EDS spectrum highlights the presence of Ag (86.13%) and fewer amounts of other elements such as O (4.54%) and Cl (9.29%) weight % and Ag (59.27), O (19.45) and Cl (21.28) atomic %, respectively^51^.

**Figure 7:**
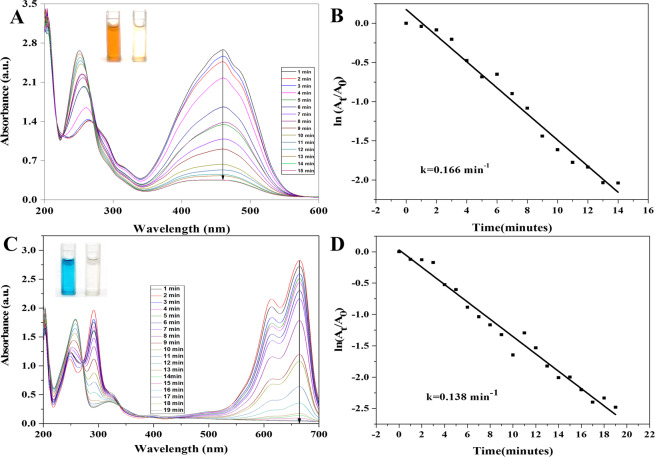
UV-visible absorption spectra of catalytic degradation of MO (**A**) and MB (**C**) by NaBH4 in the presence of AgNPs, Pseudo-first order plot of ln(At/A0) vs. time of MO (**B**) and MB (**D**).

AgNPs-mediated catalytic reduction of MB, MO, CR and 4-NP was carried out in the presence of NaBH_4_ as reducing agent. MO, a hazardous azo-dye to the environment^52^, can be degraded by reducing agent like NaBH_4_ but the rate of degradation is very low. AgNPs with the properties of low volume to high surface area can increase the rate of reduction for dye degradation. An aqueous solution of MO shows orange color which turns into deep orange by adding NaBH_4_ and it shows strong UV spectral band in the visible range at 464 nm. There was no change in absorbance of MO in the presence of NaBH_4_^53^. After adding AgNPs in MO containing NaBH_4_ the reduction in absorbance was observed shown in Fig. 7A. The degradation was analyzed by UV-visible spectrophotometer at 1 min time interval. The complete degradation of the MO dye was done within 14 min. Figure 7B shows the reduction reaction followed the pseudo-first-order reaction kinetic with the rate constant (k) 0.166 min^−1^.

The degradation of MB was achieved using *T. arjuna* derived AgNPs. MB is a cationic heterocyclic aromatic dye which is used as redox indicator in analytic chemistry, chemotherapeutic and anti-malarial agent in the aquaculture industry. An aqueous solution of MB is profound blue coloured and shows the maximum UV absorption peak at 664 nm with a bulge at 613 nm due to n → π^*^ and π → π^*^ transitions. In the presence of potent reducing agent NaBH_4_ no degradation was observed. The reduction of MB to leucomethyleneblue (colorless MB) by AgNPs with NaBH_4_ and the decrease in absorbance was monitored spectrometrically at 664 nm Fig. 7C. The reduction reaction was completed within 20 min and the reaction kinetics followed the pseudo-first-order and k = 0.138 min^−1^ is rate constant. Figure 7D.

**Figure 8:**
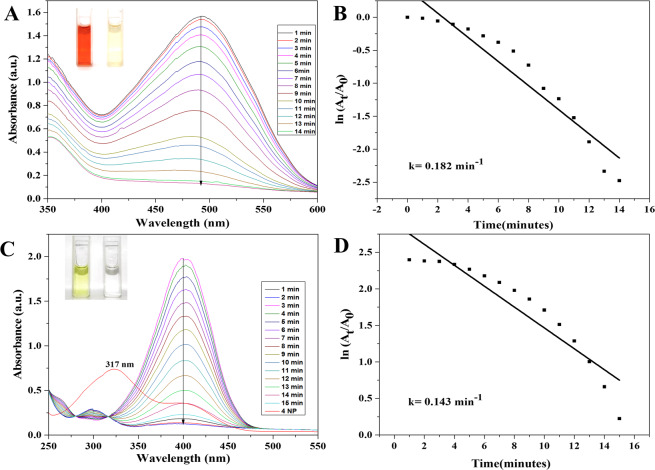
UV-visible absorption spectra of catalytic degradation of CR (**A**) and 4-NP (**C**) by NaBH4 in the presence of AgNPs, Pseudo-first order plot of ln(At/A0) vs. time of CR (**B**) and 4-NP (**D**).F.

**Figure 9:**
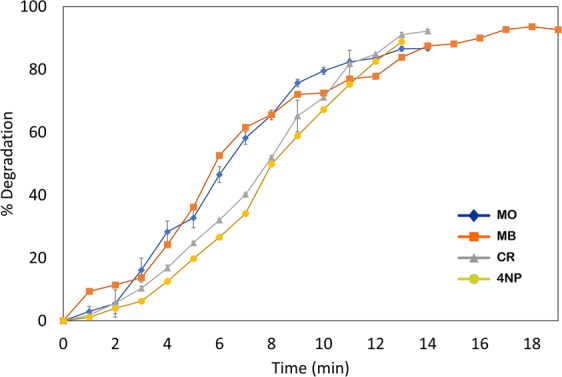
Percent degradation of organic dyes (MO, MB, CR, 4NP) with time by AgNPs.

The maximum absorption peak of the aqueous solution of CR (brick red color) and 4-NP (yellow color) was observed at 490 nm (Fig. 8A) and 317 nm (Fig. 8C) respectively. However, addition of NaBH_4_ shifted the absorption peak of 4-NP to 400 nm. The shifting of adsorption peak may be due to the formation of 4-nitrophenolate ion (Fig. 8C)^54^. The intensity of absorption remained constant and no decoloration was observed by adding NaBH_4_ for long time. Though, after adding of AgNPs to the aqueous solution of CR and 4-NP contained NaBH_4,_ instantly sharp reduction in the absorption takes place, was examined by UV spectra shown in Fig. 8A,C. The drop at 400 nm and concurrently rises at 297 nm confirms the degradation of 4-NP through the reduction of nitrophenolate to aminophenol ion (Fig. 8C). CR completely degraded within 14 min, while 4-NP took 15 min for complete degradation. The catalytic reaction kinetics and rate constant can be calculated by pseudo-first-order rate kinetic law from the plot ln(A_t_/A_0_) versus reaction time with respect of CR and 4-NP^55,56^ shown in Fig. 8B,D. The rate constant was estimated for CR and 4-NP to be 0.182 min^−1^ and 0.142 min^−1^, respectively. The percent degradation of the dye was calculated using equation (2) that shows the more than 86% of degradation was observed. In the Fig. 9 illustrated the percent degradation of MO (86.68%), MB (93.60%), CR (92.20%) and 4NP (88.80%). High volume to surface ratio of the synthesized AgNPs provides more catalytic sites and lower activation energy. Thus, the catalytic degradation of organic dyes might be because of surface reaction between reactant and AgNPs as suggested by Langmuir-Hinshelwood model^57^.

**Figure 10:**
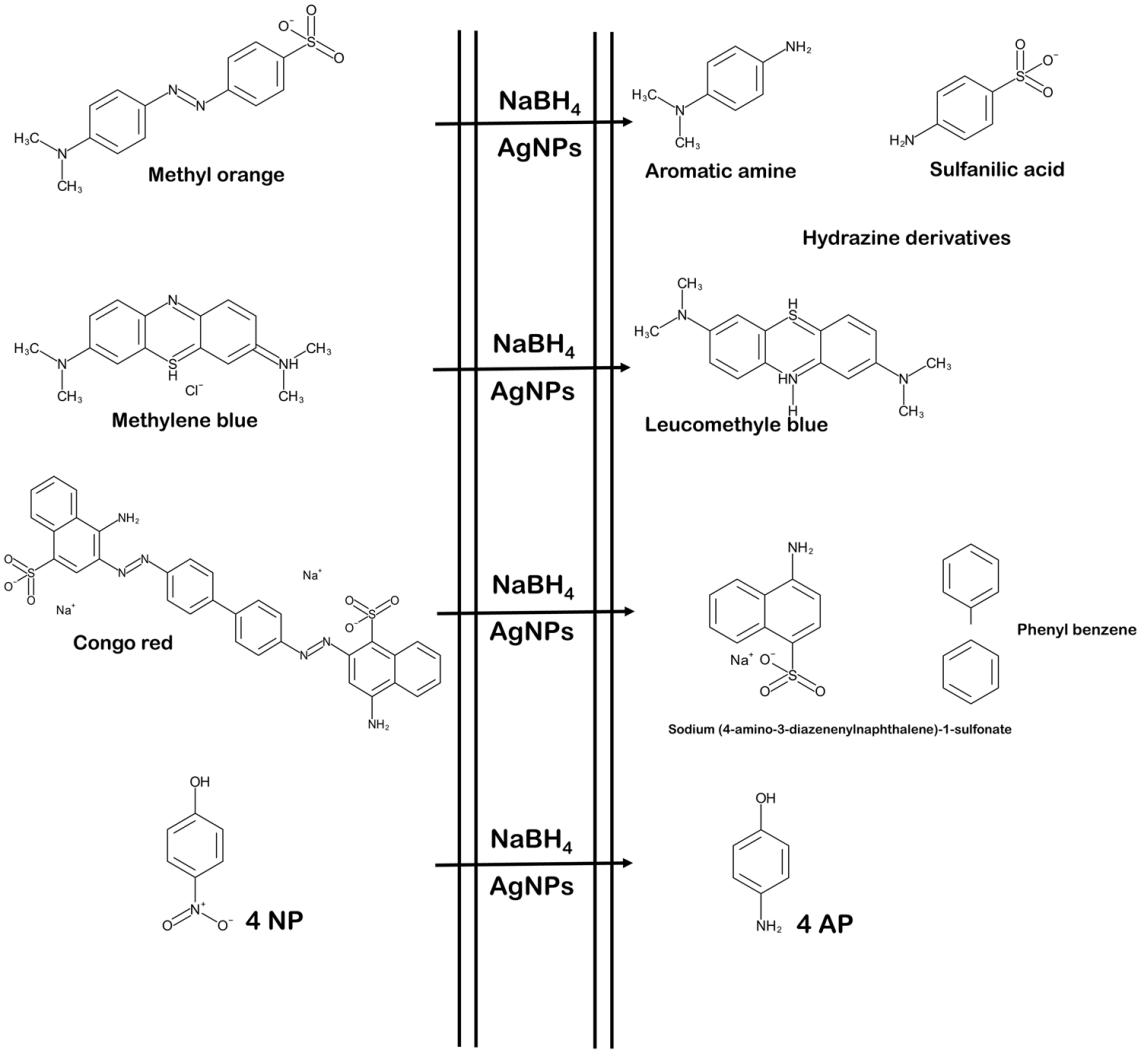
Schematic representation of reaction of dye degradation using NaBH_4_ and AgNPs.

For the reduction of several organic dyes use of NaBH_4_ as reducing agent is kinetically favourable but not thermodynamically viable. Hence various nanocatalyst have been reported to reduction of organic dyes accomplished in very less time and kinetically feasible. Figure 10 represent the schematic diagram of dye degradation. In the mechanism of catalytic reduction of organic dyes, the role of NaBH_4_ is to produce BH_4_^−1^ by its dissociation and act as electron donor and dyes molecules act as electron acceptor while AgNPs act as relay centre for electron transfer from BH_4_^−1^ ion to dyes molecules. In aqueous solution both BH_4_^−1^ and dyes molecules are diffuse to the surface of AgNPs. The BH_4_^−1^ and dyes molecule are attached to NPs surface. The hydrogen released from BH_4_^−1^ act as hydrogen source, get adsorbed by catalytic surface of NPs and attack on dyes molecules. The electron carrying NPs activate catalytic surface and activate azo bond of dyes resulting breakage of azo bonds due to uptake of electron via catalyst and hydrogen from BH_4_^−1^ and dyes get reduced on the surface of NPs^30,58^.

## Conclusion

In this study, we have reported a facile and green approach to biosynthesis of AgNPs employing *T. arjuna* leaf extract as a reducing and stabilizing agent. The synthesized AgNPs was confirmed by characterization with FTIR, UV-vis, DLS, SEM-EDS, TEM and XRD analyses. TEM and FE-SEM studies shown the particles were spherical, face cantered cubic in nature and size range in 10–50 nm. Compare to the chemical method this method is convenient and eco-friendly since it does not require any toxic chemical and surfactant. The synthesized AgNPs were successfully applied for rapid catalytic degradation of harmful organic dyes (MB, MO, CR and 4-NP). The degradation of dyes was done in less than 20 min, observed by UV-vis spectroscopic analysis. The current study provides new perspectives for biological synthesis of metal NPs and their applications as catalyst in context of wastewater treatment to remove dyes contamination from industrial effluents.

### Data analysis

The data analysis was done using Microsoft excel and the graphs were prepared using OriginPro8.5 software.

## References

[1] Ahmed S, Ahmad M, Swami BL, Ikram S (2016). A review on plants extract mediated synthesis of silver nanoparticles for antimicrobial applications: a green expertise. J. Adv. Res..

[2] Ali F, Khan SB, Kamal T, Alamry KA, Asiri AM (2018). Chitosan-titanium oxide fibers supported zero-valent nanoparticles: Highly efficient and easily retrievable catalyst for the removal of organic pollutants. Sci. Rep..

[3] Tsunoyama H, Sakurai H, Ichikuni N, Negishi Y, Tsukuda T (2004). Colloidal gold nanoparticles as catalyst for carbon-carbon bond formation: application to aerobic homocoupling of phenylboronic acid in water. Langmuir..

[4] Galletto P, Brevet PF, Girault HH, Antoine R, Broyer M (1999). Enhancement of the second harmonic response by adsorbates on gold colloids: the effect of aggregation. J. Phys. Chem. B.

[5] Maier SA (2001). Plasmonics a route to nanoscale optical devices. Adv. Mater..

[6] Han M, Gao X, Su JZ, Nie S (2001). Quantum-dot-tagged microbeads for multiplexed optical coding of biomolecules. Nat. Biotechnol..

[7] West JL, Halas NJ (2003). Engineered nanomaterials for biophotonics applications: improving sensing, imaging, and therapeutics. Annu. Rev. Biomed. Eng..

[8] Nicewarner-Pena SR (2001). Submicrometer metallic barcodes. Science.

[9] Gao Y (2005). Saturable absorption and reverse saturable absorption in platinum nanoparticles. Opt. Commun..

[10] Yanez-Sedeno P, Pingarron JM (2005). Gold nanoparticle-based electrochemical biosensors. Anal. Bioanal. Chem..

[11] Loo YY (2018). *In Vitro* Antimicrobial Activity of Green Synthesized Silver Nanoparticles Against Selected Gram-negative Foodborne Pathogens. Front. Microbiol..

[12] Lakshmanan G, Sathiyaseelan A, Kalaichelvan PT, Murugesan K (2018). Plant-mediated synthesis of silver nanoparticles using fruit extract of *Cleome viscosa* L.: Assessment of their antibacterial and anticancer activity. Karbala Int. J. Mod. Sci..

[13] Tian J (2007). Topical delivery of silver nanoparticles promotes wound healing. ChemMedChem: Chem. Enab. Drug Disc..

[14] Owoseni-Fagbenro KA (2019). Egg proteins stabilized green silver nanoparticles as delivery system for hesperidin enhanced bactericidal potential against resistant *S. aureus*. J. Drug Delivery Sci. Technol..

[15] Leopold N, Lendl B (2003). A new method for fast preparation of highly surface-enhanced Raman scattering (SERS) active silver colloids at room temperature by reduction of silver nitrate with hydroxylamine hydrochloride. J. Phys. Chem. B.

[16] Reicha FM, Sarhan A, Abdel-Hamid MI, El-Sherbiny IM (2012). Preparation of silver nanoparticles in the presence of chitosan by electrochemical method. Carbohydr. Polym..

[17] Navaladian S, Viswanathan B, Viswanath RP, Varadarajan TK (2007). Thermal decomposition as route for silver nanoparticles. Nano Res. Lett..

[18] Darroudi M, Zak AK, Muhamad MR, Huang NM, Hakimi M (2012). Green synthesis of colloidal silver nanoparticles by sonochemical method. Mater. Lett..

[19] Xia N, Cai Y, Jiang T, Yao J (2011). Green synthesis of silver nanoparticles by chemical reduction with hyaluronan. Carbohydr. Polym..

[20] Vasquez RD (2016). E.T. Polysaccharide-mediated green synthesis of silver nanoparticles from Sargassum siliquosum JG Agardh: Assessment of toxicity and hepatoprotective activity. OpenNano.

[21] Guilger-Casagrande M, Germano-Costa T, Pasquoto-Stigliani T, Fraceto LF, de Lima R (2019). Biosynthesis of silver nanoparticles employing *Trichoderma harzianum* with enzymatic stimulation for the control of *Sclerotiniasclerotiorum*. Sci. Rep..

[22] Jo JH (2016). *Pseudomonas deceptionensis* DC5-mediated synthesis of extracellular silver nanoparticles. Artif. Cell Nanomed. Biotechnol..

[23] Saravanan M, Barik SK, MubarakAli D, Prakash P, Pugazhendhi A (2018). Synthesis of silver nanoparticles from *Bacillus brevis* (NCIM 2533) and their antibacterial activity against pathogenic bacteria. Microbial pathogen..

[24] Raj S, Mali SC, Trivedi R (2018). Green synthesis and characterization of silver nanoparticles using *Enicostemmaaxillare* (Lam.) leaf extract. Biochem. Biophys. Res. Commun..

[25] Behravan M (2019). Facile green synthesis of silver nanoparticles using *Berberis vulgaris* leaf and root aqueous extract and its antibacterial activity. Int. J. Bio. Macromol..

[26] Pugazhendhi A, Prabhu R, Muruganantham K, Shanmuganathan R, Natarajan S (2019). Anticancer, antimicrobial and photocatalytic activities of green synthesized magnesium oxide nanoparticles (MgONPs) using aqueous extract of *Sargassum wightii*. J. Photochem. Photobiol. B: Biol..

[27] Kulkarni, N. & Muddapur, U. Biosynthesis of metal nanoparticles: a review, *J. Nanotech*. (2014).

[28] Dwivedi S (2007). *Terminalia arjuna* Wight &Arn.—a useful drug for cardiovascular disorders. J. ethnopharmcol..

[29] Ali SS (2008). Indian medicinal herbs as sources of antioxidants. Food Res. Int..

[30] Naseem K (2019). Catalytic reduction of toxic dyes in the presence of silver nanoparticles impregnated core-shell composite microgels. J. Clean. Prod..

[31] Rafique M (2019). Novel and facile synthesis of silver nanoparticles using *Albizia procera* leaf extract for dye degradation and antibacterial applications. Mater. Sci. Eng.: C.

[32] Vasantharaj S (2019). Synthesis of ecofriendly copper oxide nanoparticles for fabrication over textile fabrics: characterization of antibacterial activity and dye degradation potential. J. Photochem. Photobiol. B: Biol..

[33] Muthu K, Priya S (2017). Green synthesis, characterization and catalytic activity of silver nanoparticles using *Cassia auriculata* flower extract separated fraction. Spectrochim. Acta Part A.

[34] Issaabadi Z, Nasrollahzadeh M, Sajadi SM (2017). Green synthesis of the copper nanoparticles supported on bentonite and investigation of its catalytic activity. J. Clean. Prod..

[35] Vidhu VK, Philip D (2014). Catalytic degradation of organic dyes using biosynthesized silver nanoparticles. Micron.

[36] Ashraf S (2013). Protein-mediated synthesis, pH-induced reversible agglomeration, toxicity and cellular interaction of silver nanoparticles. Colloids. Surf. B.

[37] Andreescu D, Eastman C, Balantrapu K, Goia DV (2007). A simple route for manufacturing highly dispersed silver nanoparticles. J. Mater. Res..

[38] Hasan M (2018). Biological entities as chemical reactors for synthesis of nanomaterials: Progress, challenges and future perspective. Mater. Today. Chem..

[39] Martinez-Castanon GA, Nino-Martinez N, Martinez-Gutierrez F, Martinez-Mendoza JR, Ruiz F (2008). Synthesis and antibacterial activity of silver nanoparticles with different sizes. J. Nano. Res..

[40] Sun L (2008). Aggregation based growth of silver nanowires at room temperature. Appl. Surf. Sci..

[41] Gopinath K, Venkatesh KS, Ilangovan R, Sankaranarayanan K, Arumugam A (2013). Green synthesis of gold nanoparticles from leaf extract of *Terminalia arjuna*, for the enhanced mitotic cell division and pollen germination activity. Ind. crops prod..

[42] Nayak D, Ashe S, Rauta PR, Kumari M, Nayak B (2016). Bark extract mediated green synthesis of silver nanoparticles: evaluation of antimicrobial activity and antiproliferative response against osteosarcoma. Mater. Sci. Eng: C..

[43] Jiao T (2015). Reduced graphene oxide-based silver nanoparticle-containing composite hydrogel as highly efficient dye catalysts for wastewater treatment. Sci. Rep..

[44] Rout Y, Behera S, Ojha AK, Nayak PL (2012). Green synthesis of silver nanoparticles using *Ocimum sanctum* (Tulashi) and study of their antibacterial and antifungal activities. J. Microbiol. Antimicrobiol..

[45] Ajitha B, Reddy YAK, Reddy PS (2015). Green synthesis and characterization of silver nanoparticles using *Lantana camara* leaf extract. Mater. Sci Eng. C.

[46] Karuppiah M, Rajmohan R (2013). Green synthesis of silver nanoparticles using *Ixora coccinea* leaves extract. Mater. Lett..

[47] Sankar R (2013). *Origanum vulgare* mediated biosynthesis of silver nanoparticles for its antibacterial and anticancer activity. Colloids Surf. B.

[48] Bhakya S, Muthukrishnan S, Sukumaran M, Muthukumar M (2016). Biogenic synthesis of silver nanoparticles and their antioxidant and antibacterial activity. Appl. Nanosci..

[49] Francis S, Joseph S, Koshy EP, Mathew B (2017). Green synthesis and characterization of gold and silver nanoparticles using *Mussaenda glabrata* leaf extract and their environmental applications to dye degradation. Environ. Sci. Pol. Res..

[50] Kharat SN, Mendhulkar VD (2016). Synthesis, characterization and studies on antioxidant activity of silver nanoparticles using *Elephantopusscaber* leaf extract. Mater. Sci. Eng: C.

[51] Bello BA (2017). Anticancer, antibacterial and pollutant degradation potential of silver nanoparticles from *Hyphaene thebaica*. Biochem. Biophys. Res. Commun..

[52] Wang H, Li G, Jia L, Wang G, Tang C (2008). Controllable preferential-etching synthesis and photocatalytic activity of porous ZnO nanotubes. J. Phys. Chem. C.

[53] Umamaheswari C, Lakshmanan A, Nagarajan NS (2018). Green synthesis, characterization and catalytic degradation studies of gold nanoparticles against congo red and methyl orange. J. Photochem Photobiol B.

[54] Tajbakhsh M, Alinezhad H, Nasrollahzadeh M, Kamali TA (2016). Green synthesis of the Ag/HZSM-5 nanocomposite by using *Euphorbia heterophylla* leaf extract: A recoverable catalyst for reduction of organic dyes. J. Alloys Compd..

[55] Hatamifard A, Nasrollahzadeh M, Lipkowski J (2015). Green synthesis of a natrolite zeolite/palladium nanocomposite and its application as a reusable catalyst for the reduction of organic dyes in a very short time. RSC. Adv..

[56] Atarod M, Nasrollahzadeh M, Sajadi SM (2016). *Euphorbia heterophylla* leaf extract mediated green synthesis of Ag/TiO2 nanocomposite and investigation of its excellent catalytic activity for reduction of variety of dyes in water. J. Colloid Interface Sci..

[57] Wunder S, Polzer F, Lu Y, Mei Y, Ballauff M (2010). Kinetic analysis of catalytic reduction of 4-nitrophenol by metallic nanoparticles immobilized in spherical polyelectrolyte brushes. J. Phys. Chem. C.

[58] Sengan M, Veeramuthu D, Veerappan A (2018). Photosynthesis of silver nanoparticles using *Durio zibethinus* aqueous extract and its application in catalytic reduction of nitroaromatics, degradation of hazardous dyes and selective colorimetric sensing of mercury ions. Mater. Res. Bull..

